# Molecular structure, comparative and phylogenetic analysis of the complete chloroplast genome sequences of weedy rye *Secale cereale* ssp. *segetale*

**DOI:** 10.1038/s41598-023-32587-4

**Published:** 2023-04-03

**Authors:** Lidia Skuza, Piotr Androsiuk, Romain Gastineau, Łukasz Paukszto, Jan Paweł Jastrzębski, Danuta Cembrowska-Lech

**Affiliations:** 1 Institute of Biology, University of Szczecin, 71415 Szczecin, Poland; 2 Centre for Molecular Biology and Biotechnology, Institute of Biology, University of Szczecin, 71415 Szczecin, Poland; 3 Department of Plant Physiology, Genetics and Biotechnology, Faculty of Biology and Biotechnology, University of Warmia and Mazury, 10719 Olsztyn, Poland; 4 Institute of Marine and Environmental Sciences, University of Szczecin, 70383 Szczecin, Poland; 5 Department of Physiology and Biochemistry, Institute of Biology, University of Szczecin, Felczaka 3c St., 71412 Szczecin, Poland; 6 Sanprobi Sp. z o. o. Sp. k., Kurza Stopka 5c St., 70535 Szczecin, Poland

**Keywords:** Computational biology and bioinformatics, Genetics, Molecular biology, Plant sciences

## Abstract

The complete chloroplast genome of *Secale cereale* ssp. *segetale* (Zhuk.) Roshev. (Poaceae: Triticeae) was sequenced and analyzed to better use its genetic resources to enrich rye and wheat breeding. The study was carried out using the following methods: DNA extraction, sequencing, assembly and annotation, comparison with other complete chloroplast genomes of the five *Secale* species, and multigene phylogeny. As a result of the study, it was determined that the chloroplast genome is 137,042 base pair (bp) long and contains 137 genes, including 113 unique genes and 24 genes which are duplicated in the IRs. Moreover, a total of 29 SSRs were detected in the *Secale cereale* ssp. *segetale* chloroplast genome. The phylogenetic analysis showed that *Secale cereale* ssp. *segetale* appeared to share the highest degree of similarity with *S. cereale* and *S. strictum*. Intraspecific diversity has been observed between the published chloroplast genome sequences of *S. cereale* ssp. *segetale*. The genome can be accessed on GenBank with the accession number (OL688773).

## Introduction

*Secale cereale* ssp. *segetale* is one of the many species of the genus *Secale* with a previously unknown chloroplast and mitochondrial genome. However, it can be a source of desired genes (e.g., resistance to diseases, high protein content, morphological and biochemical traits) that can enrich rye or wheat breeding^1,2^. The lack of knowledge of phylogenetic relationships reduces the progress in rye breeding, which can be enriched with functional features derived from wild rye species^3^. With new biotic and abiotic stresses and climate change, there is also a need to study wild rye species, which is crucial to improving the yield and quality of this cereal^4^. Therefore, more genetic markers are needed.. One of the way to achieve this is to sequence complete chloroplast genomes. Due to their conservative and non-recombinant nature, chloroplast genomes are a solid tool in genomics and evolutionary research^5^. Certain evolutionary hotspots of the plant plastid genome, such as single nucleotide polymorphisms and insertions/deletions, may provide useful information to elucidate the phylogenetic of taxonomically unresolved plant taxa^6,7^. Thus, the availability of complete chloroplast genomes, which include new variable and informational sites, should help explain more precise phylogeny.

To participate in this effort, we have undertaken the sequencing of the complete chloroplast genomes in genus *Secale*, which are smaller and easier to analyze compared to mitochondrial genomes. So far, only the incomplete *S. cereale* cpDNA sequences (NC_021761)^8^, three sequences for *S. strictum* (KY636137, KY636138 and OL979486)^9^ and *S. sylvestre* (MW557517)^10^ are available. The chloroplast genome of *S. segetale* has recently been published^11^, however a comprehensive phylogenetic analysis based on whole chloroplast genomes has not been done to date. Therefore, we presume that analysis of the complete chloroplast genome sequences of *Secale* spp., starting with *S. sylvestre*^10^, will be useful and cost-effective for evolutionary and phylogenetic studies, as it was suggested by our previous studies^12^.

In this study, we present the complete chloroplast genome of *S. cereale* ssp. *segetale*, which will provide valuable information for genetic studies of *Secale* species.

## Results

### Chloroplast genome of *Secale cereale* ssp. *segetale*

**Figure 1:**
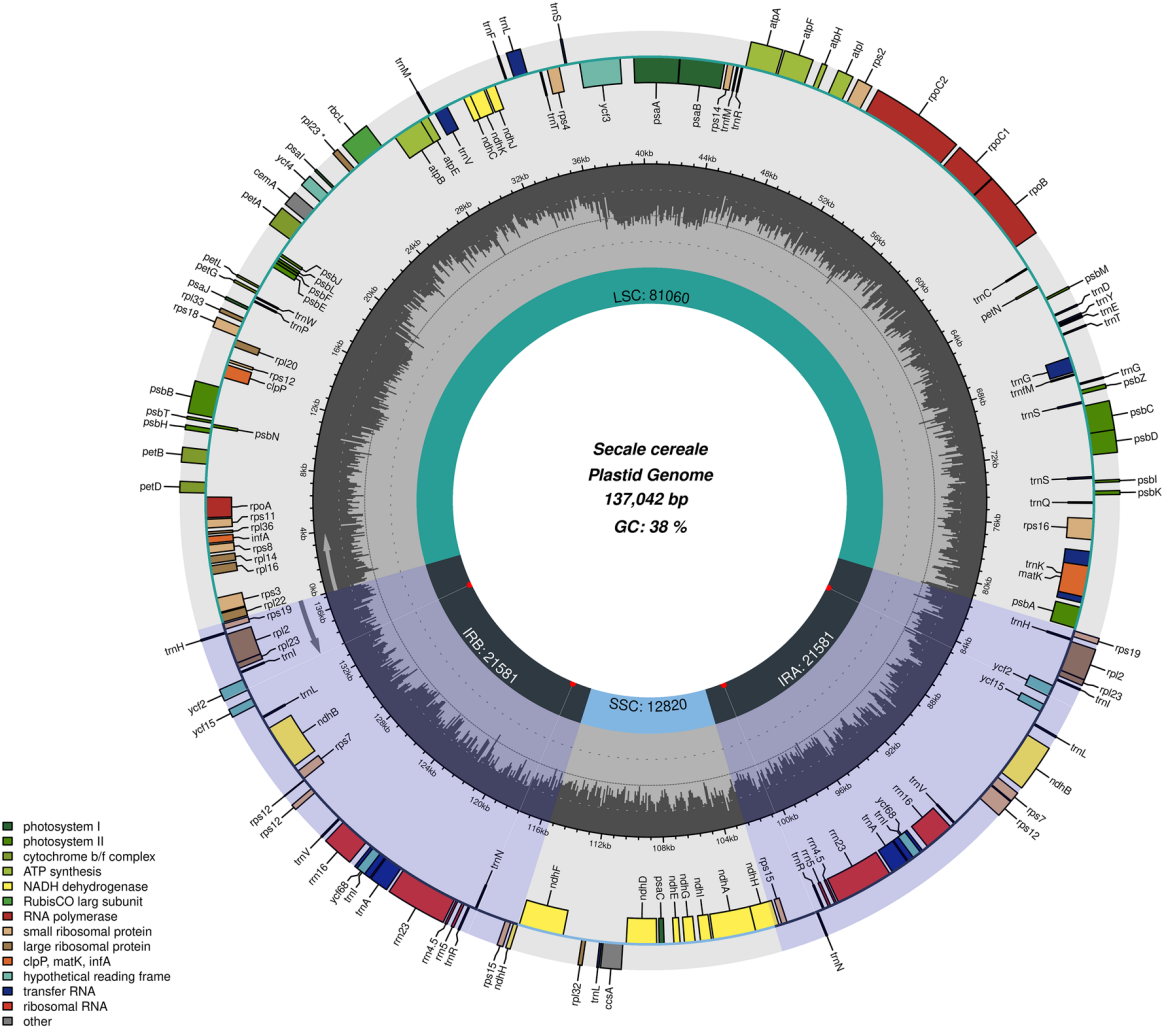
Map of the chloroplast genome of *Secale cereale* ssp. *segetale*. The genes inside and outside the circle are transcribed in the clockwise and counterclockwise directions, respectively. Genes belonging to different functional groups are shown in different colors. Tick lines indicate the extent of the inverted repeats (IRa and IRb) that separate the genomes into small single-copy (SSC) and large single-copy (LSC) regions. The innermost darker gray corresponds to GC-content while the lighter gray corresponds to AT content.

Sequencing of *Secale cereale* ssp. *segetale* chloroplast genome yielded 41 653 350 raw reads, out of which 88 777 were mapped to the reference genome of *S. cereale* with 97 × average coverage. The *S. cereale* ssp. *segetale* cp genome appeared as a typical circular, double-stranded molecule with the length of 137,042 bp (Fig. 1) and overal GC content of 38%. The large single copy (LSC) region is 81,060 bp long, the short single copy (SSC) region is 12,820 bp long, and each of the inverted repeat regions (IR) is 21,581 bp long. Reported cp genome contains 137 genes, including 113 unique genes and 24 genes which are duplicated in the IRs. Group of 113 unique genes features 73 protein-coding genes, 30 tRNA genes, four rRNA genes and five conserved chloroplast open reading frames (ORFs) (Table 1).

**Table 1:** Genes present in chloroplast genome of *Secale cereale* ssp. *segetale.* Genes list arranged alphabetically.

Category	Group of gene	Name of genes
Photosynthesis	Photosystem I	*psaA*, *psaB*, *psaC*, *psaI*, *psaJ*
	Photosystem II	*psbA*, *psbB*, *psbC*, *psbD*, *psbE*, *psbF*, *psbH*, *psbI*, *psbJ*, *psbK*, *psbL*, *psbM*, *psbN*, *psbT*, *psbZ*
	Cytochrome complex	*petA*, *petB*, *petD*, *petG*, *petL*, *petN*
	ATP synthase	*atpA*, *atpB*, *atpE*, *atpF*^*c*^, *atpH*, *atpI*
	NADH dehydrogenase	*ndhA*^*c*^^,^ *ndhB*^*c*^ (× 2), *ndhC*, *ndhD*, *ndhE*, *ndhF*, *ndhG*, *ndhH*^b^ (2x), *ndhI*, *ndhJ*, *ndhK*
	Large subunit of RUBISCO	*rbcL*
DNA replication and protein synthesis	Ribosomal RNA	*rrn4.5* (× 2), *rrn5* (× 2), *rrn16* (× 2), *rrn23* (× 2)
	Small subunit ribosomal proteins	*rps2*, *rps3*, *rps4*, *rps7* (× 2), *rps8*, *rps11*, *rps12*^e^, *rps14*, *rps15*(× 2), *rps16*^*c*^, *rps18*, *rps19* (× 2)
	Large subunit ribosomal proteins	*rpl2*^*c*^ (× 2), *rpl14*, *rpl16*, *rpl20*, *rpl22, rpl23*^b^ (× 3), *rpl32*, *rpl33*, *rpl36*
	RNA polymerase subunits	*rpoA*, *rpoB*, *rpoC1*, *rpoC2*
	Translational initiation factor	*infA*
	Transfer RNA	*trnA-UGC* ^c^(× 2), *trnC-GCA*, *trnD-GUC*, *trnE-UUC*, *trnF-GAA*, *trnfM-CAU*, trnfM-AUG, *trnG-UCC*^*c*^ (× 2), *trnH-GUG* (× 2), *trnI-CAU* (× 2), *trnI-GAU*^*c*^ (× 2), *trnK-UUU*^*c*^, *trnL-CAA* (× 2), *trnL-CUA*, *trnL-UAA*^*c*^, *trnM-CAU*, *trnN-GUU* (× 2), *trnP-UGG*, *trnQ-UUG*, *trnR-ACG* (× 2), *trnR-UCU*, *trnS-GCU*, *trnS-GGA*, *trnS-UGA*, *trnT-GGU*, *trnT-UGU*, *trnV-GAC* (× 2), *trnV-UAC*^*c*^, *trnW-CCA*, *trnY-GUA*
Other genes	Conserved hypothetical chloroplast ORF	*ycf2* (× 2), *ycf3*^*ad*^, *ycf4*^*a*^, ycf15 (× 2), *ycf68* (× 2)
	Other proteins	*ccsA*, *cemA*, *clpP*, *matK*

^a^Genes associated with Photosystem I.

^b^One copy of the gene is a pseudogene.

^c^Gene containing one intron.

^d^Gene containing two introns.

^e^Transspliced gene.

The LSC region appeared as the most abundant in genes—57 PCGs, 21 tRNA genes and two ORFs (*ycf3* and *ycf4*), whereas there are only ten PCGs and one tRNA gene in SSC. In IR there are four rRNA genes, eight tRNA genes, three ORFs (*ycf2*, *ycf15* and *ycf68*) and nine PCGs including *ndhH* located on the junction between IR and SSC region.

### Repeat sequence analysis

A total of 52 repeat sequences structures with length ranging from 30 to 286 bp were revealed in the plastome of *Secale cereale* ssp. *segetale* (Table 2). The forward repeats (37) dominated over palindromic (15) repeats. Neither complementary nor reverse repeats were found. Most repeat sequences (69.3%) were detected in the LSC region, followed by IR (28.8%) and SSC regions (1.9%). 50% of these sequences were found within coding regions. The highest number of repeats were found within the sequences of the following genes: *rpoC2* (9F), *rpl23* (2F and 2P) and *rps18* (3F and 1P).

**Table 2:** List of repeated sequences in the chloroplast genome of *Secale* *cereale* ssp. *segetale.*

Repeat length	Strat site of repeat A	Location	Repeat A region	Strat site of repeat B	Location	Repeat B region	Repeat type
286	56561	*rpl23*	LSC	83149	*rpl23*	IR	P
286	56561	*rpl23*	LSC	134665	*rpl23*	IR	F
160	56687	*rpl23*	LSC	83149	*rpl23*	IR	P
160	56687	*rpl23*	LSC	134791	*rpl23*	IR	F
74	12628	IGS (*trnG-UCC–trnfM-CAU*)	LSC	12796	IGS (*trnfM-CAU–trnG-UCC*)	LSC	F
60	101806	IGS (*trnN-GUU–rps15*)	IR	101806	IGS (*trnN-GUU–rps15*)	IR	P
60	101806	IGS (*trnN-GUU–rps15*)	IR	116234	IGS (*trnN-GUU–rps15*)	IR	F
60	116234	IGS (*trnN-GUU–rps15*)	IR	116234	IGS (*trnN-GUU–rps15*)	IR	P
45	56364	IGS (*rbcL–rpl23*)	LSC	56364	IGS (*rbcL–rpl23*)	LSC	P
42	41556	IGS (*psaA–ycf3*)	LSC	41570	IGS (*psaA–ycf3*)	LSC	F
41	12735	*trnfM–CAU*	LSC	36344	*trnfM-CAU*	LSC	F
40	12628	IGS (*trnG-UCC–trnfM-CAU*)	LSC	36407	IGS (*trnfM-CAU–rps14*)	LSC	F
40	12796	IGS (*trnfM-CAU–trnG-UCC*)	LSC	36407	IGS (*trnfM-CAU–rps14*)	LSC	F
39	12835	IGS (*trnfM-CAU–trnG-UCC*)	LSC	36447	IGS (*trnfM-CAU–rps14*)	LSC	F
39	14437	IGS (*trnG-UCC–trnT-GGU*)	LSC	89975	IGS (*rps7–trnV-GAC*)	IR	F
39	14437	IGS (*trnG-UCC–trnT-GGU*)	LSC	128086	IGS (*rps7–trnV-GAC*)	IR	P
38	38373	*psaB*	LSC	40597	*psaA*	LSC	F
36	7542	*trnS-GCU*	LSC	44850	*trnS-GGA*	LSC	P
36	43333	I intron *ycf3*	LSC	90638	IGS (*rps7–trnV-GAC*)	IR	F
36	43333	I intron *ycf3*	LSC	127426	IGS (*rps7–trnV-GAC*)	IR	P
35	12667	IGS (*trnG-UCC–trnfM-CAU*)	LSC	36447	IGS (*trnfM-CAU–rps14*)	LSC	F
35	12719	*trnfM-CAU*	LSC	36328	*trnfM-CAU*	LSC	F
35	76754	*infA*	LSC	76772	*infA*	LSC	F
34	27191	*rpoC2*	LSC	27212	*rpoC2*	LSC	F
34	27253	*rpoC2*	LSC	27328	*rpoC2*	LSC	F
33	27113	*rpoC2*	LSC	27164	*rpoC2*	LSC	F
33	61501	IGS (*petA–psbJ*)	LSC	61501	IGS (*rbcL–rpl23*)	LSC	P
32	11271	*trnS-UGA*	LSC	44857	*trnS-GGA*	LSC	P
32	12677	IGS (*trnG-UCC–trnfM-CAU*)	LSC	36457	IGS (*trnfM-CAU–rps14*)	LSC	F
32	14794	*trnT-GGU*	LSC	46100	*trnT-UGU*	LSC	P
32	27072	*rpoC2*	LSC	27171	*rpoC2*	LSC	F
32	27219	*rpoC2*	LSC	27273	*rpoC2*	LSC	F
32	41556	IGS (*psaA–ycf3*)	LSC	41584	IGS (*psaA–ycf3*)	LSC	F
31	8407	IGS (*trnS-GCU–psbD*)	LSC	8444	IGS (*trnS-GCU–psbD*)	LSC	F
31	12843	IGS (*trnfM-CAU–trnG-UCC*)	LSC	36455	IGS (*trnfM-CAU–rps14*)	LSC	F
31	15484	IGS (*trnY-GUA–trnD-GUC*)	LSC	33828	intron *atpF*	LSC	F
31	27054	*rpoC2*	LSC	27324	*rpoC2*	LSC	F
31	27064	*rpoC2*	LSC	27259	*rpoC2*	LSC	F
31	27241	*rpoC2*	LSC	27382	*rpoC2*	LSC	F
31	27316	*rpoC2*	LSC	27382	*rpoC2*	LSC	F
31	66279	*rps18*	LSC	66300	*rps18*	LSC	F
31	80266	*rps3*	LSC	80311	*rps3*	LSC	F
31	101837	IGS (*trnN-GUU–rps15*)	IR	116265	IGS (*trnN-GUU–rps15*)	IR	F
31	105588	IGS (*ndhF–rpl32*)	SSC	105612	IGS (*ndhF–rpl32*)	SSC	F
30	12870	*trnG-UCC*	LSC	36314	*trnfM-CAU*	LSC	F
30	16756	IGS (*psbM–petN*)	LSC	16756	IGS (*psbM–petN*)	LSC	P
30	66231	*rps18*	LSC	66315	*rps18*	LSC	F
30	66298	*rps18*	LSC	66319	*rps18*	LSC	F
30	66677	*rps18*	LSC	66677	*rps18*	LSC	P
30	87638	Intron *ndhB*	IR	87638	Intron *ndhB*	IR	P
30	87638	Intron *ndhB*	IR	130432	Intron *ndhB*	IR	F
30	130432	Intron *ndhB*	IR	130432	Intron *ndhB*	IR	P

IGS (*trnG-UCC–trnfM-CAU*) means spacer between *trnG-UCC* and *trnfM-CAU.*

A total of 29 SSRs were detected in the *Secale cereale* ssp. *segetale* chloroplast genome (Table 3). The mononucleotide SSRs composed of A/T units were the most common, whereas hexanucleotide SSRs were not detected. 79.3% of SSRs were located within LSC region, 13.8% in IR region while only 6.9% of SSRs were found in SSC region. Most of the SSRs were identified within intergenic spacers (58.6%), while equal proportions (20.7%) were located in the introns and coding sequences.

**Table 3:** Distribution of SSR in the *Secale cereale* ssp. *segetale* cp genome.

Type	Repeat unit	Length	Start	End	Location	Region
Mononucleotide	A	13	7211	7223	IGS (*psbK–psbJ*)	LSC
		13	7937	7949	IGS (*trnS-GCU–psbD*)	LSC
		18	11227	11244	IGS (*psbC–trnS-UGA*)	LSC
		12	29538	29549	*rpoC2*	LSC
		12	29945	29956	IGS (*rpoC2–rps2*)	LSC
		12	33569	33580	intron *atpF*	LSC
		12	33844	33855	intron *atpF*	LSC
		13	36192	36204	IGS (*trnR-UCU–trnfM-CAU*)	LSC
		12	43027	43038	II intron *ycf3*	LSC
		13	46728	46740	IGS (*trnT-UGU–trnL-UAA*)	LSC
		12	76716	76727	IGS (*rpl36–infA*)	LSC
		12	105202	105213	IGS (*ndhF–rpl32*)	SSC
Trinucleotide	AAT	15	24652	24666	*rpoC1*	LSC
	AAT	13	47511	47523	IGS (*trnL-UAA–trnF-GAA*)	LSC
	AAC	12	31552	31563	*atpI*	LSC
	AAT	12	56494	56505	IGS (*rbcL–rpl23*)	LSC
	AAG	12	65670	65681	IGS (*psaJ–rpl33*)	LSC
Tetranucleotide	AAGG	12	42927	42938	II intron ycf3	LSC
	AATG	12	64604	64615	IGS (*trnW-CCA–trnP-UGG*)	LSC
	AAAG	12	64889	64900	IGS (*trnP–UGG-psaJ*)	LSC
	AAAG	12	68899	68910	IGS (*clpP–psbB*)	LSC
	AACG	13	99471	99483	*4.5S rRNA*	IR
	AAAT	12	108269	108280	*ndhD*	SSC
	AACG	13	118618	118630	*4.5S rRNA*	IR
Pentanucleotide	AATAT	18	15656	15673	IGS (*trnY-GUA–trnD-GUC*)	LSC
	ACCAT	15	43805	43819	I intron *ycf3*	LSC
	AATAT	18	47154	47171	intron *trnL-UAA*	LSC
	AATAT	16	101098	101113	IGS (*trnN-GUU–rps15*)	IR
	AATAT	16	116988	117003	IGS (*trnN-GUU-rps15*)	IR

IGS IGS (*psbK-psbJ*) means spacer between *psbK* and *psbJ.*

### Multigene phylogeny

**Figure 2:**
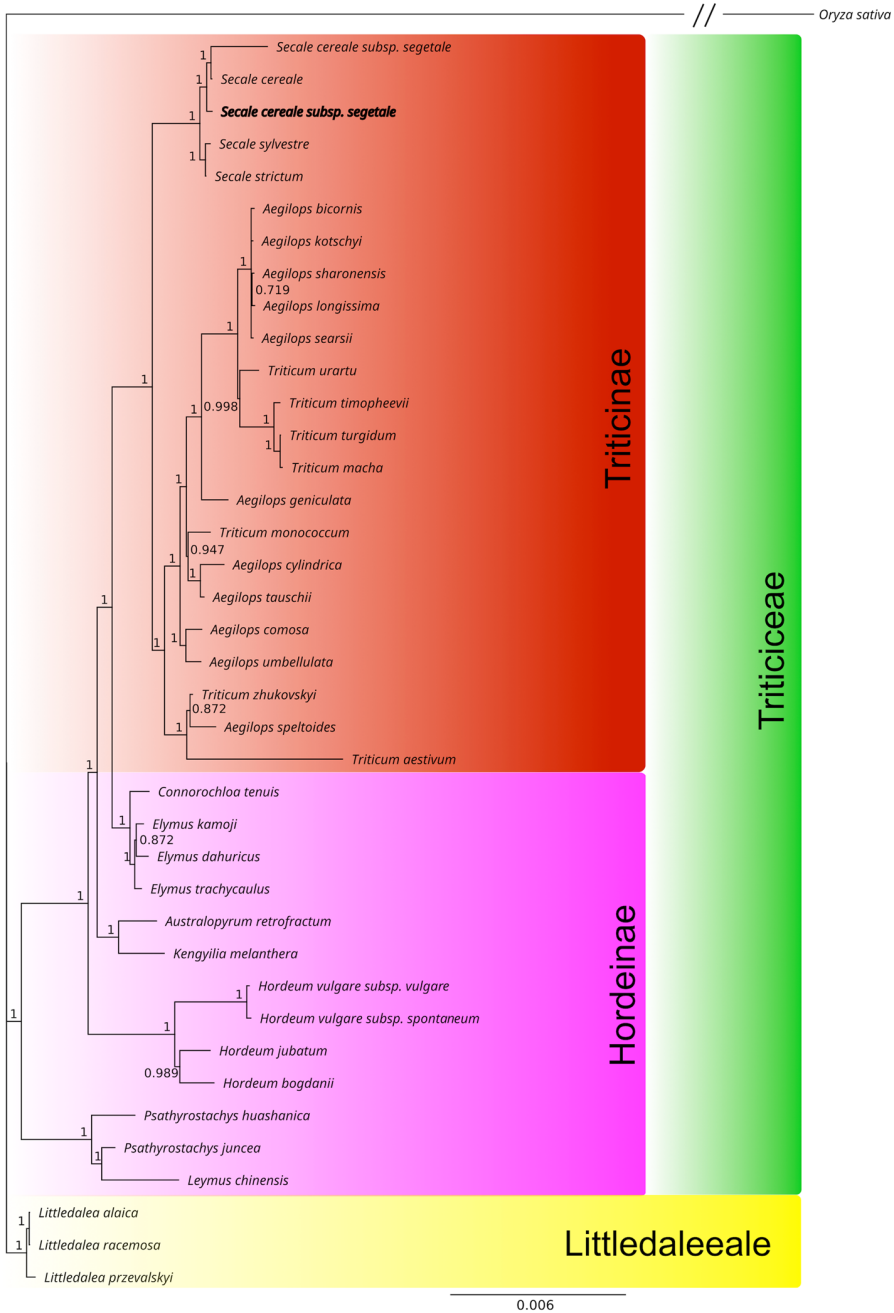
Cladogram illustrating the phylogenetic relationships for *Secale cereale* ssb*. segetale* based on complete cp genome sequences. Phylogenetic tree based on sequences of sheared 73 protein-coding genes from five *Secale* species and 34 other cereal lineages representing Triticodae group within subfamily Pooidae and the cp genome of *Oryza sativa* as an outgroup, using Bayesian posterior probabilities (PP) and maximum likelihood (ML). Each node has 100% bootstrap support value. The cpDNA sequence obtained in this study is shown in bold.

Phylogeny reconstruction based on sequences of 73 protein-coding genes shared by *Secale cereale* ssp. *segetale* and 38 representatives of Pooideae subfamily appeared to be consistent with the systematic position of studied species. The BI and ML tree divided analyzed species into six major clades (Fig. 2). The first cluster contained 23 species representing Triticinae subtribe, four other clades gathered 13 species representing Hordeinae subtribe, whereas the last clad consisted of three *Littledalea* species (Littledaleeae tribe). *Secale cereale* ssp. *segetale* appeared to share the highest degree of similarity with *S. cereale* and *S. cereale* ssp. *segetale*. Mentioned above five *Secale* species form separate sub-clad within the Triticinae tribe.

### Comparison with other complete chloroplast genomes of the *Secale* species

**Figure 3:**
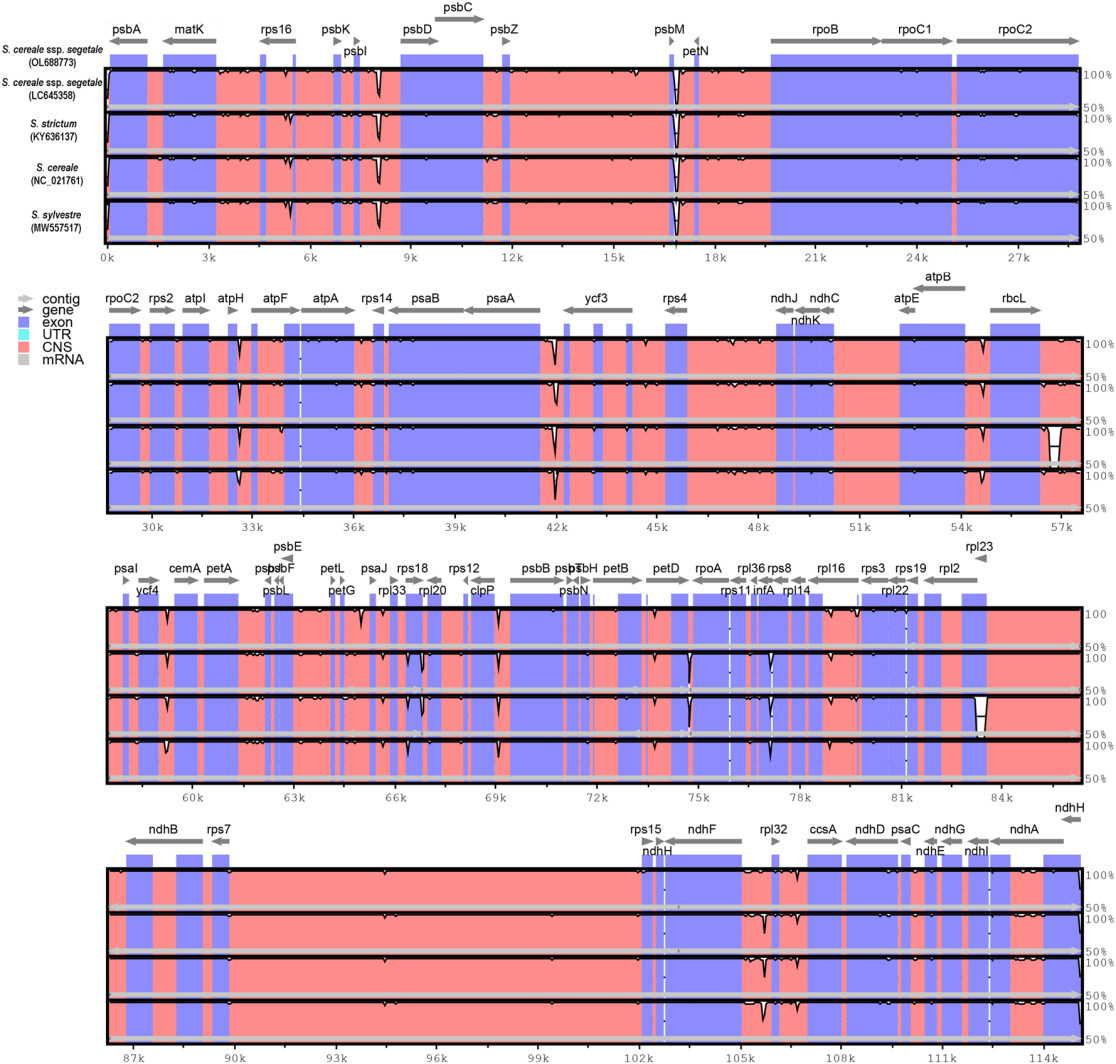
Percentage of sequence identity between chloroplast genomes of *Secale cereale* ssp. *segetale* and other four *Secale* species using mVISTA program. Gray arrows on the top line show transcriptional direction. The y-axis represents average percent identity between sequences of *S. cereale* ssp*. segetale* and other three *Secale* chloroplast genomes. The x-axis represents the coordinate in the chloroplast genome using *S. cereale* ssp. *segetale* as reference. Genome regions are color coded as exon, untranslated regions (UTR), and conserved non-coding sequences (CNS).

The overall sequence identity of five cp genomes of *Secale* species was plotted using mVISTA with the annotation of *S. cereale* ssp. *segetale* cp genome (obtained by new sequencing in this study) as reference (Fig. 3). The results showed that the *Secale* cp genomes exhibited a high level of sequence synteny, suggesting a conserved evolutionary pattern. The plastome sequences were fairly conserved across the four data with a few regions with a variation. The sequences of exons were nearly identical throughout the all taxa.

## Discussion

The task of modern cereal breeding is to obtain new, higher-yielding varieties that have high resistance to pathogens, diseases and abiotic conditions. Unfortunately, progress in rye breeding has been limited, as the varieties used in cultivation have had limited variability due to selection. In addition, attempts to use old varieties have been unsuccessful.

A major advance in rye breeding has been the introduction of hybrid varieties, through which individual genotypes are fixed by continuing self-pollination and transferring monogenic traits into varieties^13^. However, despite the increase in yield, intermediate quality traits are subject to large annual fluctuations. Thus, despite significant increases in grain yield and decreases in protein content in the experiments, increases in grain yield did not significantly positively or negatively affect intermediate quality traits^4^.

A number of taxa in the genus *Secale* may represent a potential source of genetic variability in rye breeding^3^. Species such as *Secale strictum* and *Secale vavilovii* may be sources of new genetic variability, with resistance to ear fusariosis and septoria leaf blotch), while *Secale vavilovii* may also be a source of sterilizing cytoplasm (source of sterilising cytoplasm). Wild rye species and subspecies provide excellent starting material for studies aimed at expanding recombination variability in cultivated rye and triticale (× Triticosecale Wittmark). Because of their genetic distinctiveness and high trait expression, they represent a valuable source of genes in which our cultivars are deficient^14^. An example is the study of the efficiency of crossing the wild species *Secale vavilovii* and the rye subspecies *Secale cereale* ssp. *afghanicum*, *Secale cereale* ssp. *ancestrale*, *Secale cereale* ssp. *dighoricum*, *Secale cereale* ssp. *segetale* with the crop species *Secale cereale* ssp. *cereale*, and the resulting F1 crosses may be a potential source of variation in common rye^3^. Unfortunately, the lack of knowledge of phylogenetic relationships reduces the progress in rye breeding.

For understanding plant origin and evolution chloroplast genome sequences are very useful. With maternally inherited traits, a genome of relatively small size and a slow mutation rate of the genome^15^, analysis of the phylogenetic relationships of multiple chloroplast DNA can help understand plant phylogeny, population genetic analysis and taxonomic status at the molecular level^16^.

Although cp genomes of angiosperm plants are generally conservative in terms of sequence and number of genes^17^, levels of structural variation have been observed in the genome that vary across families and genera, such as gene duplication and large-scale rearrangements of genes, introns and IR domains (e.g.^18,19^).

The *S. cereale* ssp. *segetale* cp genome appeared as a typical circular, double-stranded molecule (Fig. 1) and overal GC content, which is similar to previously sequenced plastomes of *S. cereale* (137,051 bp; NCBI LC645358), *S. sylvestre* (137 116 bp)^10^ or within the size range of angiosperms^20^.

The results obtained by Du et al.^11^ are similar to ours. The size of the genome, the lengths of the LSC, SSC and IR sequences differ slightly. In contrast, larger differences are seen in the number of genes. The genome we analyzed contains 73 protein-coding genes (82 in^11^), 30 tRNA genes (41 in^11^) four rRNA genes (8 in^11^) and five conserved chloroplast open reading frames (ORFs)(lack of information in^11^).

It is difficult to say where the above-mentioned differences came from. The rich interspecific genetic diversity of *S. S. cereale* ssp. *segetale* has been previously reported (e.g.^21^). Significant differences were found between and within populations of *S. c.* ssp. *segetale*. A high degree of genetic variability has also been described using chromosomal markers^22,23^. These results deserve attention and further research.

The polymorphisms found in *S. c.* ssp. *segetale* chloroplast genome sequences can be used e.g. to elucidate evolutionary histories such as the origin of *Secale* species or accessions at the inter- and, thanks to the research described in this manuscript, intra-species level. Furthermore, the polymorphic sites promote practical applications for molecular analysis to protect *S. c.* ssp. *segetale* accession^24^ and, potentially in the long term, the rye breeding industry. Unfortunately, the analyses of the genome previously published by Du et al. do not include many details, in addition to those mentioned above, which does not allow for a more detailed analysis.

Certain regions of the plastome are predisposed to indel and substitution mutations. Comparative studies of the plastome show the evolution of, among other, tandem repeats and their role in generating substitutions and indels^25,26^. Once the composition of repeat sequences in the plastome is determined, it is possible to predict microstructural changes by analyzing the correlation between repeats, indels and substitutions. In addition to the paucity of genomic resources, the phylogeny of the genus *Secale* is enigmatic (e.g.^27,28^). Therefore, it is important to fully explore the polymorphic regions of *Secale* chloroplasts in an evolutionary context.

For the total of 52 repeat sequence structures revealed in the *Secale* *cereale* ssp. *segetale* plastome, the vast majority were detected in the LSC region (Table 2). The highest number of repeats was found within the sequences of the *rpoC2*, *rpl23* and *rps18* genes. Regardless of its function the *rpoC2,* gene encoding the β-subunit of plastid RNA polymerase is a relatively rapidly evolving chloroplast sequence^29^. Analogically, *rpl23* gene and its pseudogene which are observed in the grass family belong to highly polymorphic genes considered as a hotspots of illegitimate recombination in cp genomes^30^.

Chloroplast SSRs identification not only serves as a one of cp genome characteristics but also represent ideal molecular tools with various applications like investigation of domestication history, sites of origin or genetic diversity and relationships between wild and cultivated species^31,32^. In 2016, Hagenblad et al.^33^ analyzed the genetic diversity of 76 accessions of wild, feral and cultivated rye based on SNP polymorphisms. They performed an analysis of five chloroplast SSRs, derived from *Lolium* and wheat. Discriminant analysis of principal components (DAPC) of cpSSR data indicated very large genetic variation within the genus *Secale* and did not reflect taxonomic groups, except for *S. strictum* and *S. africanum*, which formed a separate cluster.

CpSSRs are mainly distributed within intergenic spacers of *Secale* plastomes; similar distribution preferences of cpSSRs have been reported in *Avena* spp., P*seudoroegneria libanotica* and *Salvia miltiorrhiza*^34–36^.

Phylogenetic analysis has shown that *Secale cereale* ssp. *segetale* appeared to share the highest degree of similarity with *S. cereale* and *S. cereale* ssp. *segetale*. The five *Secale* species form separate sub-clad within the Triticinae tribe, which confirms previous phylogenetic data of the genus *Secale* (e.g.^37^).

The results showed that the *Secale* cp genomes exhibited a high level of sequence synteny, suggesting a conserved evolutionary pattern. The plastome sequences were fairly conserved across the four data with a few regions with a variation. The sequences of exons were nearly identical throughout the all taxa.

## Conclusions

Here we assembled the complete, annotated chloroplast genome sequence of *Secale cereale* ssp. *segetale*. The genome is 137 042 base pair (bp) long and contains 137 genes, including 113 unique genes and 24 genes which are duplicated in the IRs. The phylogenetic analysis showed that *Secale cereale* ssp. *segetale* appeared to share the highest degree of similarity with *S. cereale* and *S. strictum*. Intraspecific diversity has been observed between the published chloroplast genome sequences of *S. cereale* ssp. *segetale*. The cp genome will provide a series of resources for evolutionary and genetic studies about species of rye. The assembled genome sequences and annotation information have been deposited in GenBank under the accession number OL688773.

## Material and methods

### DNA extraction, sequencing, assembly and annotation

Seeds of *Secale cereale* ssp. *segetale* introd. no. 1782/94 were obtained from the Botanical Garden of the Polish Academy of Sciences in Warsaw. Total DNA was extracted from young sprouts following Doyle and Doyle^38^.

The chloroplast (cp) genome of *Scecale* *cereale* ssp. *segetale* was sequenced with the use of DNBseq platform in BGI Shenzhen (China). After the quality check (FastQC tool available online at: http://www.bioinformatics.babraham.ac.uk/projects/fastqc) the raw reads were mapped to the reference genome of *Secale* *cereale* (NC_021761) in Geneious v.R7 software with default medium–low sensitivity settings^39^. Reads aligned to the reference cpDNA genome were extracted and used for de novo assembly (K-mer—23–41, low coverage cut-off—5, minimum contig length—300). De novo contigs were extended by mapping raw reads to the generated contigs, reassembling the contigs with mapped reads, and manually scaffolding the extended contigs (minimum sequence overlap of 50 bp and 97% overlap identity). This process was iterated five times. Finally, the reduced sequences were assembled in the circular chloroplast genome. The chloroplast genome was annotated using MFannot^40^ and PlasMapper^41^ with manual adjustments. The gene map of the annotated cp genome was developed with the OrganellarGenome DRAW tool^42^.

### Repeat sequence analysis

The chloroplast simple sequence repeats (SSRs) were detected using Phobos v.3.3.12^43^. Only perfect SSRs with a motif size of one to six nucleotide units were considered, the following thresholds for chloroplast SSRs identification were used: ≥ 12 repeat units for mononucleotide SSRs, ≥ 6 repeat units for dinucleotide SSRs, ≥ 4 repeat units for trinucleotide SSRs, and ≥ 3 repeat units for tetra-, penta- and hexanucleotide SSRs^44^. Analysis of long genomic repeats, i.e. forward (F), reverse (R), palindromic (P) and complementary (C) sequences, was performed using REPuter software^45^ with the following settings: (1) hamming distance of 3, (2) sequence identity ≥ 90%, and (3) minimum repeat size ≥ 30 bp. A single IR region was used to eliminate the influence of doubled IR regions.

### Multigene phylogeny

The phylogenetic position of *Scecale* *cereale* ssp. *segetale* within Triticodae group was also evaluated. For that purpose 73 concatenated protein-coding gene sequences shared with other 38 Pooideae species were used. The cpDNA of *Oryza sativa* was used as an outgroup (Table 4). For phylogeny reconstruction Bayesian Inference (BI) method was used. The best-fit model of sequence evolution was identified in MEGA v.7^46^, and the GTR + G + I model was selected. The BI analysis was performed in MrBayes v.3.2.6^47^. Parameter settings were previously described by Androsiuk et al.^48^.

**Table 4:** List of species used in phylogenetic studies. Species names arranged alphabetically.

Species	Accession number
*Oryza sativa*	NC_008155
*Aegilops bicornis*	NC_024831
*Aegilops comosa*	NC_046697
*Aegilops cylindrica*	NC_023096
*Aegilops geniculata*	NC_023097
*Aegilops kotschyi*	NC_024832
*Aegilops longissima*	NC_024830
*Aegilops searsii*	NC_024815
*Aegilops sharonensis*	NC_024816
*Aegilops speltoides*	NC_022135
*Aegilops tauschii*	NC_022133
*Aegilops umbellulata*	NC_046696
*Australopyrum retrofractum*	NC_043840
*Connorochloa tenuis*	NC_037165
*Elymus dahuricus*	NC_049159
*Elymus kamoji*	NC_051511
*Elymus trachycaulus*	NC_050404
*Hordeum bogdanii*	NC_043839
*Hordeum jubatum*	NC_027476
*Hordeum vulgare *subsp.* Spontaneum*	NC_042692
*Hordeum vulgare *subsp.* vulgare*	NC_008590
*Kengyilia melanthera*	NC_042706
*Leymus chinensis*	NC_044900
*Littledalea alaica*	NC_037519
*Littledalea przevalskyi*	NC_037497
*Littledalea racemosa*	NC_036350
*Psathyrostachys huashanica*	NC_045871
*Psathyrostachys juncea*	NC_043838
*Secale cereale*	NC_021761
*Secale cereale* subsp*. segetale*	LC645358
*Secale cereale* subsp*. segetale*	LC645358
*Secale strictum*	KY636137
*Secale sylvestre*	MW557517
*Triticum aestivum*	NC_002762
*Triticum macha*	NC_025955
*Triticum monococcum*	NC_021760
*Triticum timopheevii*	NC_024764
*Triticum turgidum*	NC_024814
*Triticum urartu*	KJ614411
*Triticum zhukovskyi*	NC_046698

For multigene phylogeny maximum likelihood (ML) analyses was conducted using RAxML-NG^49^ under three different strategies. (1) One of the IR regions was removed from all chloroplast genomes to reduce overrepresentation of duplicated sequences then we run RAxML-NG on the unpartitioned alignment under GTR + I + G substitution model as a single partition; (2) The same data was partitioned by gene, exon, intron and intergenic spacer regions and allowed separate base frequencies, α-shape parameters, and evolutionary rates to be estimated for each; (3) we inferred the best-fitting partitioning strategy with PartitionFinder2^50^ for the alignment. The best fitting nucleotide substitution models were inferred with jModelTest2^51^. Phylogenetic trees were visualized and edited with FigTree 1.4.4^52^. Support for the ML tree branches was calculated by the non-parametric bootstrap method with 1000 replicates.

### Comparison with other complete chloroplast genomes of the *Secale* species

The percentage of sequence identity among complete chloroplast genomes of the five *Secale*: *S. cereale* ssp. *segetale* (OL688773), *S. cereale* ssp. *segetale* (LC645358), *S. cereale* (NC_021761), *S. strictum* (KY636137), and *S. sylvestre* (MW557517) was comparatively analyzed and plotted using the program mVISTA^53^, with alignment algorithm of LAGAN^54^, a cut-off of 70% identity, and annotation of *S. cereale* ssp. *segetale* (OL688773) as reference.

### Ethics approval and consent to participate

Authors confirm that the use of plants in the present study complies with international, national and/or institutional guidelines.

## Data Availability

The genome can be accessed on GenBank with the accession number (OL688773).
